# Swiss-PO: a new tool to analyze the impact of mutations on protein three-dimensional structures for precision oncology

**DOI:** 10.1038/s41698-021-00156-5

**Published:** 2021-03-18

**Authors:** Fanny S. Krebs, Vincent Zoete, Maxence Trottet, Timothée Pouchon, Christophe Bovigny, Olivier Michielin

**Affiliations:** 1 Computer-Aided Molecular Engineering, Department of Oncology, Ludwig Institute for Cancer Research Lausanne Branch, University of Lausanne, Lausanne, Switzerland; 2 Molecular Modelling Group, Swiss Institute of Bioinformatics (SIB), Lausanne, Switzerland; 3 Department of Oncology, Ludwig Institute for Cancer Research, University Hospital of Lausanne, Lausanne, Switzerland

**Keywords:** Cancer, Structural biology

## Abstract

Swiss-PO is a new web tool to map gene mutations on the 3D structure of corresponding proteins and to intuitively assess the structural implications of protein variants for precision oncology. Swiss-PO is constructed around a manually curated database of 3D structures, variant annotations, and sequence alignments, for a list of 50 genes taken from the Ion AmpliSeq^TM^ Custom Cancer Hotspot Panel. The website was designed to guide users in the choice of the most appropriate structure to analyze regarding the mutated residue, the role of the protein domain it belongs to, or the drug that could be selected to treat the patient. The importance of the mutated residue for the structure and activity of the protein can be assessed based on the molecular interactions exchanged with neighbor residues in 3D within the same protein or between different biomacromolecules, its conservation in orthologs, or the known effect of reported mutations in its 3D or sequence-based vicinity. Swiss-PO is available free of charge or login at https://www.swiss-po.ch.

## Introduction

Facilitated access to next-generation sequencing (NGS) has triggered the development of precision oncology programs around the world for tumors escaping standard therapies^1^. Decision-making is often centralized in molecular tumor boards (MTB) that regroup experts from various disciplines needed for a thorough analysis of the tumor specimen’s anatomo-pathological features, its genomics landscape obtained by NGS gene panels, as well as the precise clinical context of the patient. The number of nonsynonymous somatic mutations found in tumors varies greatly^2,3^, but gene panels covering between 50 and 500 genes typically yield several dozen variants to be analyzed. Most of these variants are of unknown significance and only a small fraction is actionable driver mutations. Deciphering passenger vs. driver mutation is, therefore, of paramount importance to guide therapy. In addition, a driver mutation might alter the inhibitory profiles of known drugs. Estimating the impact of such mutation on the activity of the various drugs at disposal is critically important to support decision-making in precision oncology. Finally, as integration of all this information is essential, live discussions in preparation or during the MTB represent an important added value as it allows challenging and contextualizing results. To address these important aspects, we have designed Swiss-PO, the first interactive tool to assess the structural implications of protein variants for precision oncology.

Predicting the influence of a missense, nonsense, deletion, insertion, duplication, or frameshift mutation on the structure of a protein requires access to the three-dimensional (3D) structure of the protein to apprehend the role of the native residue on the structural stabilization of one of its domains, on the binding of a substrate, cofactor or drug, or on the interaction with another domain or another biomacromolecule. This information can be extracted from the Protein Data Bank^4^. The importance of the role of a residue can also be appreciated by analyzing its conservation among orthologs. It is thus necessary to retrieve the corresponding protein sequences and to align them. Ultimately, the interpretation of the predicted structural impact on the activity of the protein can be facilitated by knowledge of the experimentally determined effect of other mutations of the same protein. These data can be retrieved from many databases, such as ClinVar^5^, ClinGen^6^, CIViC^7^, cBioPortal^8^, OncoKB^9^, PMKB^10^, Uniprot/Swiss-Prot^11^, CKB Core and Boost^12^, and SVIP-O^13^ for instance. To enable the molecular modeling-based interpretation of mutational effects, we have grouped this information on one single website called Swiss-PO (swiss-po.ch).

Other websites have been developed to map gene mutations or known clusters of mutational hotspots on the 3D structure of corresponding proteins, including COSMIC-3D^14^, CRAVAT^15^, 3DHotspots.org^16^, MOKCA^17^, MuPIT^18^, Cancer3D^19^, LS-SNP/PDB^20^, SNP2Structure^21^, MutationAssessor^22^, and G23D^23^. Generally, they display proteins without permitting detailed analysis between the mutated residue and its surroundings. Swiss-PO differs from these tools in that it has been designed from the ground up to enable the analysis of molecular interactions involving the protein residues, to understand the effect of potential mutations of several types as long as they can be mapped on a 3D structure (missense, nonsense, deletion, insertion, duplication, frameshift, or insertion of a stop codon). To this end, all structure files extracted from the PDB for the proteins of interest were manually curated to (i) collect information on the quality of the structure; (ii) renumber the residues in accordance with the UniProt reference sequence when necessary; (iii) note the presence of ligands of interest, notably U.S. Food and Drugs Administration (FDA) approved drugs; (iv) identify the protein domains present in the structure, as well as the residue coverage, mutations and intra or extracellular localization; (v) characterize the state of the protein, e.g., if a kinase is in its active or inactive state; and (vi) determine the presence of biologically relevant biomacromolecules in complex with the protein of interest. The latter is particularly important, for instance, if the protein is a receptor, an enzyme operating on another macromolecule, or if its activity is controlled by its binding to one or several other proteins. All this information is provided to the users, and is also used to rank the structural files from the most informative to the less relevant. This ranking facilitates the choice of the most appropriate structure to analyze regarding the mutated residue, the role of the protein domain it belongs to or the drug that could be selected to treat the patient. As an example, the PDB contains 80 structural files for the human BRAF kinase alone. Guiding the users toward the one to analyze in priority through rational criteria is a major advantage provided by Swiss-PO. Contrarily to other tools, Swiss-PO has been conceived to show not only the position of the mutated residue in 3D, but also to display all surrounding residues from the same protein or other bound proteins and domains, and to show their interactions with the wild type residue of interest in terms of hydrogen bonds, ionic, π or nonpolar interactions, etc. This information is essential to qualitatively predict the effect introduced by the mutation on the structural stability of the protein or on its interactions with a drug or some protein partners. To facilitate this analysis, Swiss-PO also provides annotations regarding the effects of known mutations that have been determined experimentally. This information is dynamically linked to the 3D display, to advise users if residues present in the vicinity of the mutated one are themselves known to be important for the structure and activity of the protein, and therefore to provide clues regarding the possible effect of a structural perturbation in this region (e.g., if reported mutations in the vicinity are activating, inactivating, or neutral). Finally, Swiss-PO also displays sequence alignments between the human proteins and selected orthologs, to provide information regarding sequence conservation of the residues of interest that can be related to their importance for protein structure and activity. Again, for a given protein, the sequence alignment is dynamically linked to the 3D display and table of variant annotations, to facilitate the simultaneous analysis of the reported effect of the mutations of a given residue, and of the interactions with its 3D surroundings. All information is displayed on a single web page, to empower a smooth and straightforward analysis of a given mutation. Importantly, Swiss-PO has been designed to be used by oncologists, pathologists, or biologists to facilitate their work in precision oncology.

## Results and discussion

### Databases preparation

**Fig. 1:**
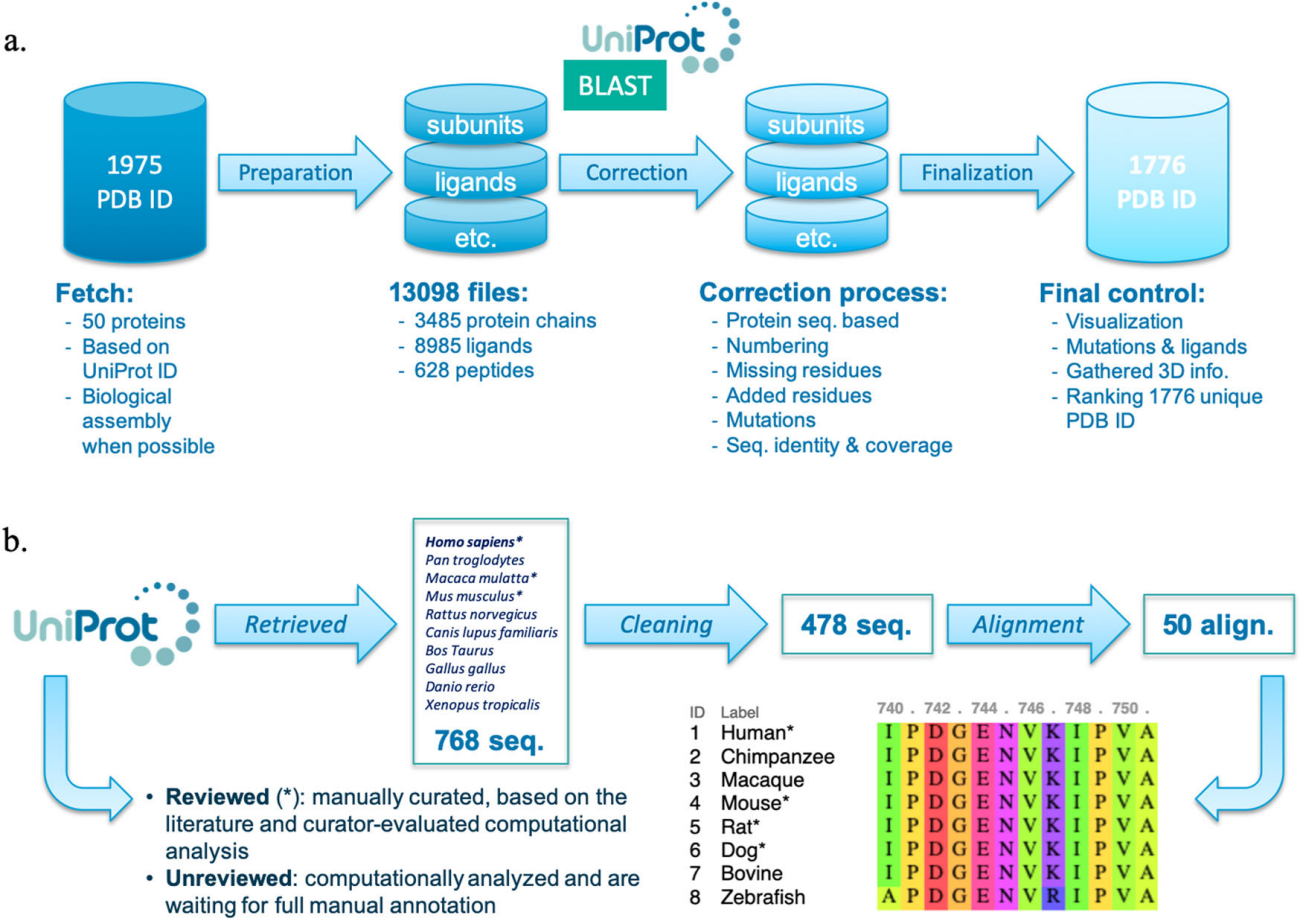
Databases preparation. **a** Sequence database preparation. **b** 3D-structure database preparation. (1) 3D structures were fetched from the PDB using their unique human UniProt code and parsed based on their chains. (2) Correction and control of the composition of each chain. (3) Final controls including visualization and ranking of the structures.

The Swiss-PO website is built on a manually curated database extracted from reliable sources such as the UniProt/Swiss-Prot^11^, the PDB^4^, and the CKB CORE database^24^. We created the database of the first release of Swiss-PO based on the Ion AmpliSeqTM Custom Cancer Hotspot Panel from Thermo Fisher covering 218 hotspots in 50 genes^25^, leading to our 50-panel list, Supplementary Table 1. The database contains two main components: one dedicated to multiple sequence alignments and the other to the 3D structures (Fig. 1).

#### Known mutation database

Knowledge regarding the experimentally determined impact of known mutations on protein structure and activity was retrieved from the CKB CORE database, with the exception of proteins DDR2, EGFR, and PIK3CA whose data were generously provided by The Jackson Laboratory from the CKB BOOST database. These data were supplemented by variant annotations provided by the Swiss-Prot group of the SIB Swiss Institute of Bioinformatics.

#### 3D-structure database

For our 50-panel list of genes, 1975 3D structures were fetched from the PDB database based on their unique human UniProt code (last update April 17, 2020), to avoid retrieving structures where the name of a gene corresponds to the acronym of a completely different one. For example, *CDH1* is the gene name of protein cadherin-1 and it can also refer to the fizzy-related protein homolog, whose more common name is *FZR1*. A name search for CDH1 in the PDB leads to a list of structures where some are related to FZR1, such as 5L9T or 5L9U. Even using this safe protocol, exceptions appeared on many occasions due to nonusual or nonstandard annotations in the header of the PDB, which describes structural information (for example, protein description, structure composition such as the presence of ligands, sequence engineering introducing mutated residues, crystallographic method, citations, etc.). For each complex, when possible, the biological assembly was retrieved in the quaternary form corresponding to what has been demonstrated or believed to be the functional form of the molecule or complex. A structure is often composed of several amino acid chains, originating from the same or different organisms, together with small molecules. First, for all the 1975 structures retrieved from the PDB, we checked and extracted amino acid chains, ligands, and peptides, which led to a total of 13,098 files (Supplementary Table 2), corresponding to 3485 protein chains, 8985 ligands including 47 FDA and 41 investigational ones (Supplementary Tables 3 and 4) for a total of 1051 unique ones, and 628 peptides (without UniProt code) and antibodies (define as such in the PDB file) including 207 unique ones. In the ligand section, we included ions, sugars, water, buffer compounds, and other small molecules. The protein chain section, which corresponds to the apo form of each chain, is composed of protein originating from proteins belonging to our 50-panel list, plus proteins that were found in complexes.

Due to resolution limitations or regions of high flexibility, some residues are not resolved and are missing in the final published 3D structures. For the sake of having the most complete structures, based on the first and last residue of the X-ray structure and BLAST sequence alignment (see Supplementary Data file), we kept only chains that have fewer than 30% missing residues compared to the similar region of their reference sequence. This step allows finding residue insertions, as well as mutated and missing residues that are unmentioned in the original PDB annotations. This information is added to the corresponding information file of Swiss-PO and is mentioned in the informative 3D-structure table of the visualization section. Thereafter, residue numbers present in the coordinate and information files of each chain related to a protein of the 50-panel list were renumbered according to their corresponding human sequence protein by considering mutated, missing, or inserted residues. This step ensures that the residue numbering in the 3D structures displayed in Swiss-PO is identical to the one of the human sequence selected from UniProt, used as a reference. When it was possible to have access to the protein information, this process was also applied to structural chains corresponding to proteins that are not in the 50-panel list but are present in 3D structures of their complexes. Handling of structure files of protein complexes is detailed in Supplementary Data file.

For each PDB file, we gathered structural information including the number and nature of the chains, the method used for its determination (e.g., X-ray diffraction, neutron diffraction, NMR solution, fiber diffraction, cryo-electron microscopy), experimental quality metrics (e.g., resolution, R-work and R-free values), the coverage of the sequence (in percentage and by residues, including gaps), as well as the mutated, missing, and added residues. Besides, we stored information regarding the subunit chains description and their UniProt code when applicable, the ligands ID and their usual name if they are FDA approved drugs or experimental drugs and the presence of sugars bound to glycosylation positions on proteins. In addition, we also created a remark container to save data relative to the domain of the target structure that is present in the structure file and, when possible, more specific information such as if kinase domains are in an active or inactive conformation. The position of the mutations and missing residues mentioned in the PDB header was checked, and unmentioned modifications were reported. Each structure was visually inspected to verify that it is correctly renumbered, following the selected human sequence. We also checked the relevance of ligands and hid irrelevant ones (for example, buffer molecules). Structures with more than ten mutations were removed, as well as the ones that are judged irrelevant, redundant, or with a wrong numbering due to an alignment failure. Note that we made an exception for gene *GNA11* for which a structure with more than ten mutations was selected as it was the only one available.

Finally, all 3D structures, for each protein, were scored according to the rules listed in Supplementary Table 5, to rank them based on their relevance, so as to allow users to easily find the most appropriate one for their analysis. The principle of the scoring is detailed in Supplementary Data file. Of note, no experimental structure was available for gene MPL.

#### Sequence curation and multiple sequence alignments

For each gene of our 50-panel list, the human amino acid sequence was retrieved from the UniProt database for the related protein. The amino acid sequences of the same protein in nine other organisms were also collected. Some of these organisms were selected for their proximity to *Homo sapiens*, such as chimpanzee and macaque, while others were taken from different taxonomic groups present in vertebrates (mouse, rat, dog, bovine, chicken, zebrafish, and frog) (Supplementary Table 6). This selection of species was inspired by the National Center of Biotechnology Information reference sequence organisms that are used for comparative analysis of sequences. The diversity of the selected organisms provides valuable information regarding the conservation of a particular residue or region, helping to evaluate the potential impact of its mutation. The most relevant sequence for each species was retained based on the protocol described in Supplementary Data file.

For each gene, the selected sequences, human and orthologous ones, were aligned with MUSCLE^26^, and the resulting alignments were checked. Important gaps were annotated, as well as particular information such as if a gene is present as a pair that derives from the same ancestral gene but resides at different locations within the same genome (for example, *ERBB4a* and *ERBB4b* in zebrafish), or, as mentioned before, when an unreviewed sequence was preferred to a reviewed one. All this information is summarized in a table under the alignment section of the Swiss-Po web page. The reviewed sequences are marked with an asterisk (*) close to their corresponding organism’s name. The preparation of the sequence alignments for the 50 genes required the curation of 768 sequences, which reduced to 478 curated ones (Supplementary Table 7). Note that for some genes, the amino acid sequences of certain organisms are missing because no corresponding sequence was found in the UniProt database (last update May 22, 2019) (see Supplementary Information for more information).

### Website

#### Graphical interface

**Fig. 2:**
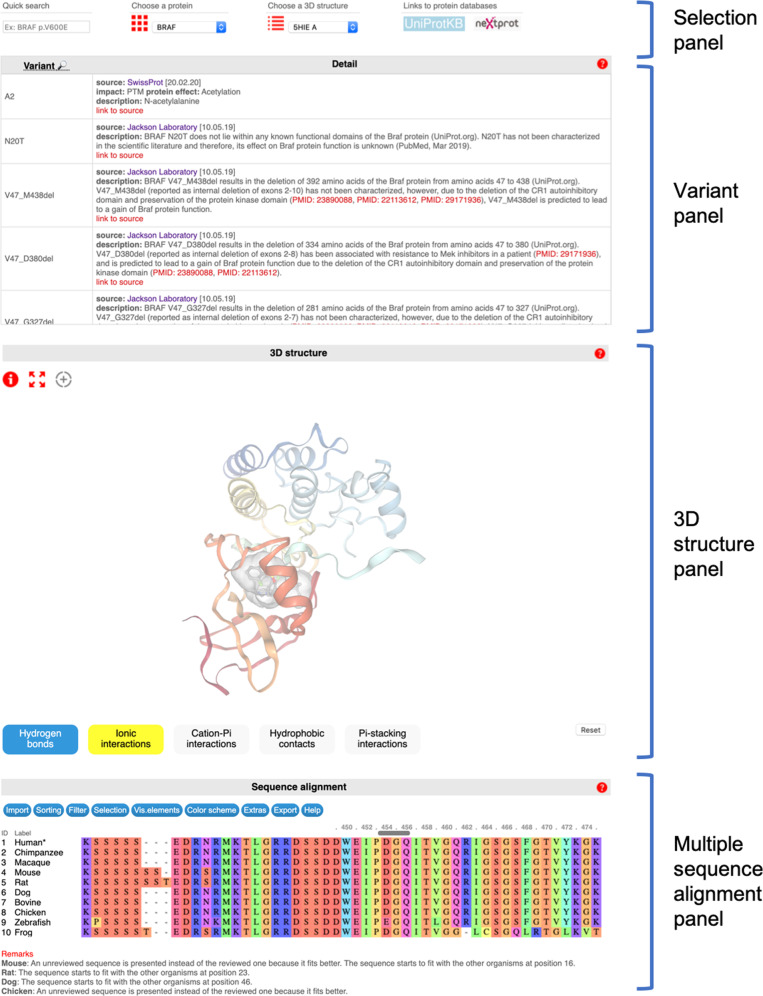
General structure of the website. Definition and position of the four panels.

The main web page is composed of four panels (Fig. 2): (i) a selection panel to choose the protein of interest and the 3D structure to display, (ii) a variant panel which lists the effect of known mutations of the selected protein, (iii) a 3D-structure panel which allows displaying a chosen 3D structure of the protein and analyzing the interactions made by the wild type residue of interest, and finally (iv) a multiple sequence alignment panel to display the alignment of the sequences of the human protein and well-chosen orthologs.

Of note, to facilitate the analysis of a given mutation, the variant, 3D structure, and sequence alignment panels are dynamically linked: selecting a residue on one of these panels will automatically focus the two others on the same residue. A more detailed description is provided below.

Each of the last three panels is accompanied by a help button, in the form of a question mark icon on the right side of the panel header, which opens a pop-up description of the main commands.

#### The selection panel

**Fig. 3:**
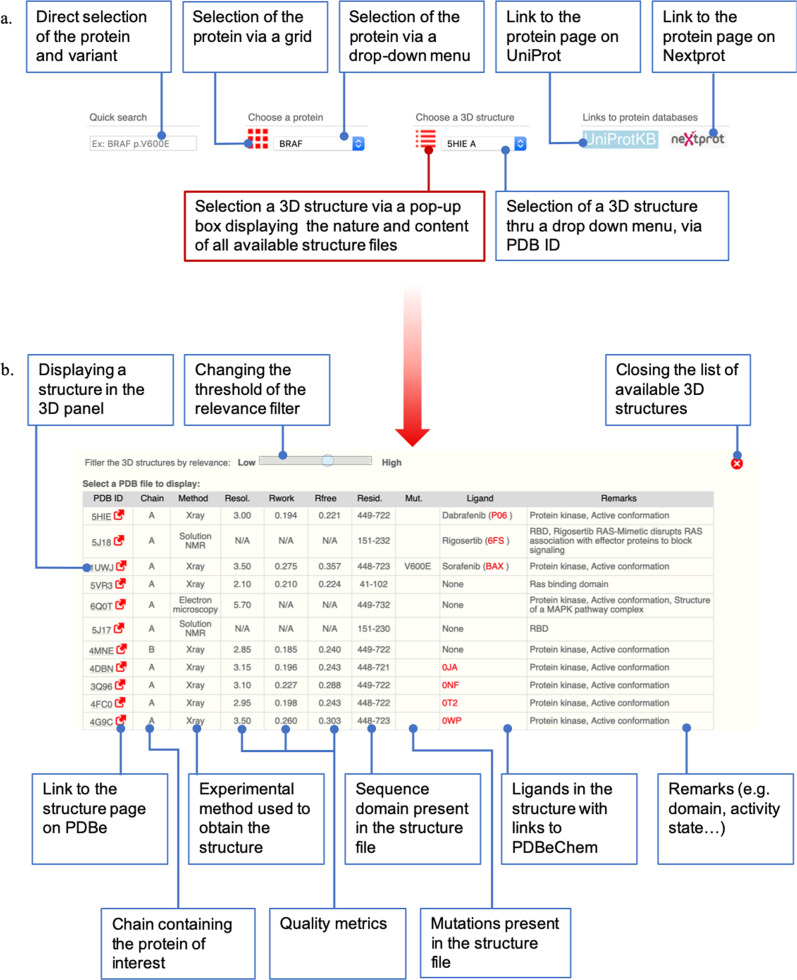
The selection panel. **a** Description of the selection panel. **b** Description of the structure list table.

The selection panel allows selecting the protein of interest and the 3D structure to display when at least one is available (Fig. 3). The choice of the protein can be performed via a drop-down menu listing the macromolecules in alphabetical order or through a grid. Once the protein has been selected, the most relevant structure, as determined by the score described above and Supplementary Table 5, is automatically displayed by default in the 3D-structure panel. Users can display other structures. The latter can be selected from a drop-down menu introduced for advanced users who already know which 3D structure they would like to display and want to pick it rapidly. Selection can also be made through a table which lists, for each available structure, information regarding the protein domain and ligands present in the PDB file, as well as the resolved residues and possible mutations. The table also reports the experimental method used to obtain the 3D structure and quality measures. By default, the table will only display the top scored, i.e., the most relevant structures (Supplementary Table 5). However, more structures can be displayed in this table by changing the threshold of the relevance filter from “high” to “low” (Fig. 3). This table allows selecting the most appropriate 3D structure based on the location of the mutation of interest, the content of the structures, and their quality. Clicking on the four-character PDB code in the left-hand column opens the corresponding structure in the 3D-structure panel. A link to the corresponding entry in the PDBe database^27^ is also provided in the left-hand column. Ligands potentially present in 3D structures are listed according to their usual name if they are approved or experimental drugs or following the three-character coding of the PDB otherwise. In both cases, a link is provided to the PDBeChem ligand dictionary to provide more information on this small molecule. Other options for the selection panel are described in Supplementary Data file.

#### The variant panel

The interpretation of the impact of a mutation on a protein structure and activity can be guided by knowledge regarding the experimentally determined consequences of other reported mutations affecting the same residue(s) or other residues situated in the vicinity in terms of sequence or 3D structure.

**Fig. 4:**
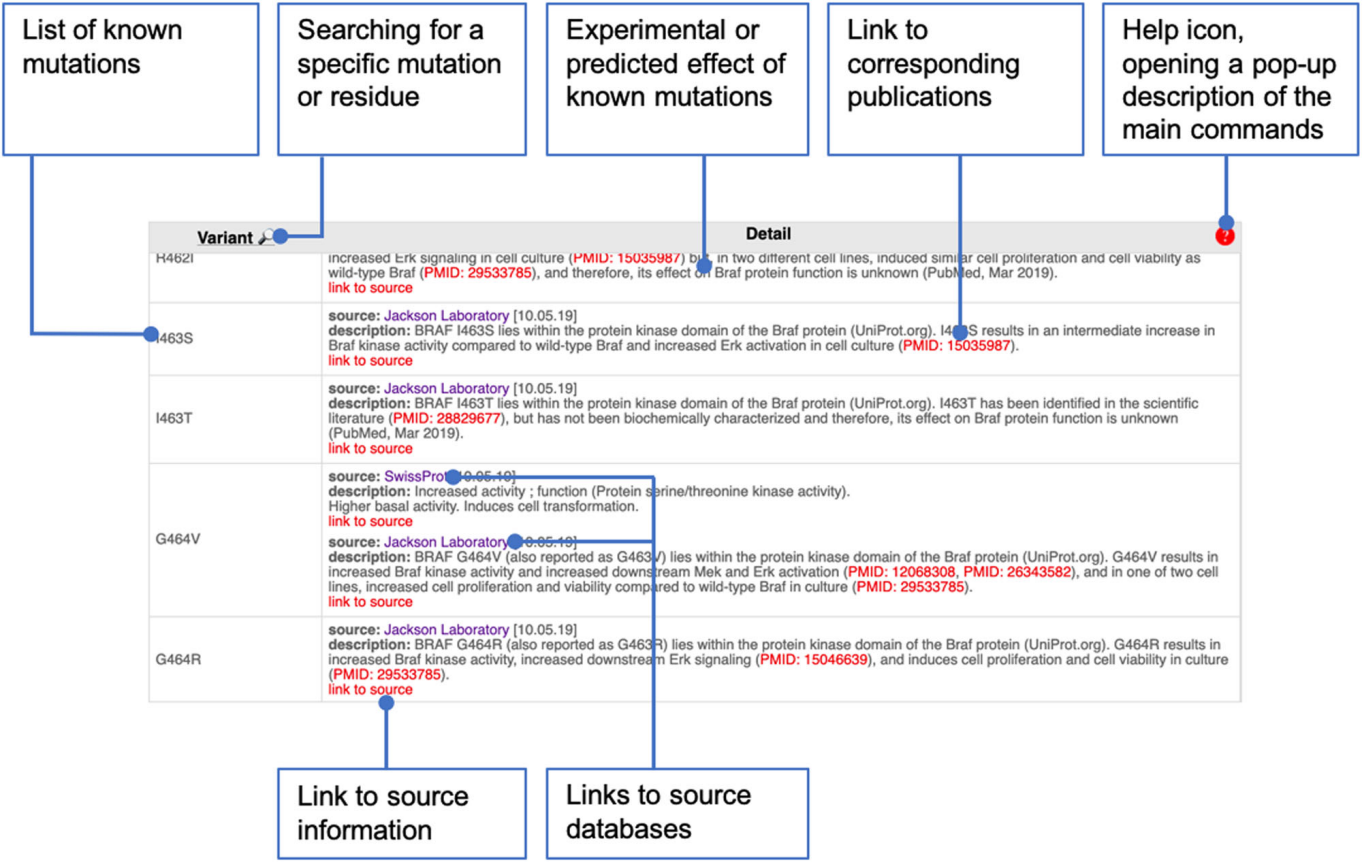
Variant panel description. The panel provides information regarding known variants. In the current version, the data was taken from the CKB Core and the Swiss-Prot databases.

To facilitate this process, we retrieved known variants of the 50 genes from the CKB CORE and BOOST databases, as well as from the Swiss-Prot database. These variants, along with their known impact on the protein structure and activity, manually curated by The Jackson Laboratory and Swiss-Prot are tabulated in the variant panel (Fig. 4). Importantly, a double click on a mutation label in the “Variant” left column of this table will focus on the 3D-structure viewer (if the residue is present in the selected 3D structure) and the sequence alignment of orthologs accordingly. The mutations impacting residues present on the displayed 3D structure are given with a white background, while others are shaded.

The “Detail” column lists the information available for each mutation, along with the source of the data (The Jackson Laboratory or Swiss-Prot) and links to the original description of the source databases as well as to relevant publications, when available.

Since mutations can also impact the protein structure and activity by influencing posttranslational modifications, the latter were retrieved from UniProt/Swiss-Prot to be provided in the variant panel. Other options for the selection panel are described in Supplementary Data file.

#### The 3D-structure panel

**Fig. 5:**
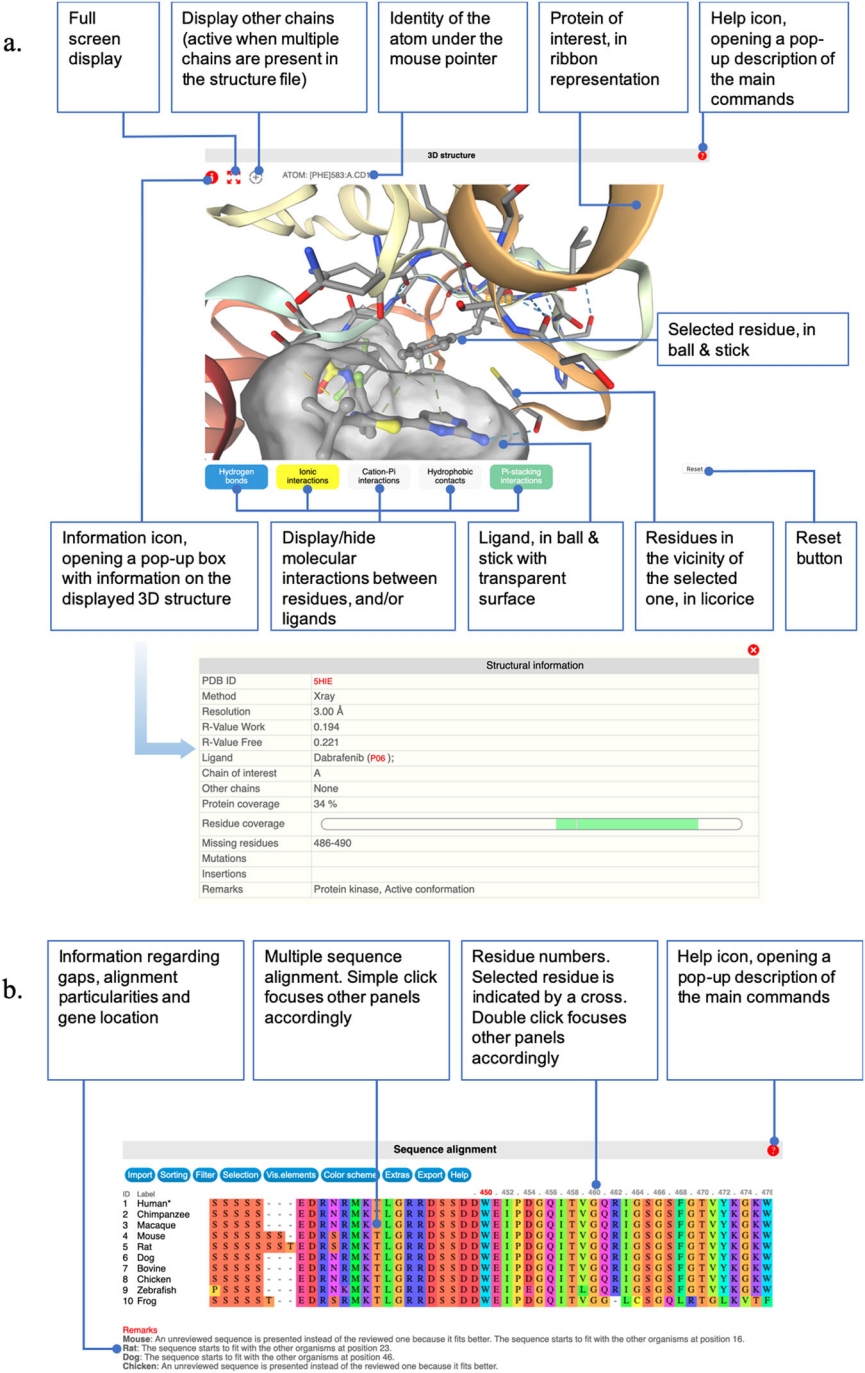
3D-structure and sequence alignment panels. **a** 3D-structure panel. **b** Multiple sequence alignment panel.

The 3D-structure panel constitutes the central component of the Swiss-PO main web page, dedicated to the analysis of the possible effect of a mutation on the protein structure and activity (Fig. 5a). As described above, important efforts were invested in exhaustively retrieving all 3D structures of interest for our selection of 50 proteins, manually curating and correcting them and identifying the most relevant ones to provide users with a ready-to-use collection of structures of particular interest for the analysis. By default, the 3D-structure panel is filled with the top-ranked 3D structure, which is considered as one of the most relevant structure (Supplementary Table 5). Other structures can be displayed upon selection of the most appropriate ones for the user’s needs. The main chain of the protein of interest is displayed in ribbon representation, colored according to the residue number, from red for the N-terminus to blue for the C-terminus. Important ligands, identified during the manual curation of the PDB file, are displayed in ball and stick representation, colored according to the atom types, and surrounded by a transparent surface.

A double click on a residue or a ligand centers the view on it, while displaying it in ball and stick and the close neighbors in stick. In parallel, if the double click is done on a residue of the main chain, the table of variants and the sequence alignment of the orthologs will focus on the same residue. A single click on a residue will center the system on it, without changing the selection of residues displayed in ball and stick or licorice.

Importantly, it is possible to display several types of molecular interactions between the selected residue or ligand and its environment using the buttons available below the 3D viewer: hydrogen bonds, ionic interactions, cation–π interactions, hydrophobic contacts, and π-stacking interactions. These interactions are displayed as dotted lines connecting the involved atoms. The color coding of the interaction types in the 3D display follows the one of the buttons that command their display or hiding.

By default, only the main chain, corresponding to the protein of interest, is displayed in the 3D-structure panel. However, impacts of mutation are sometimes originating from their effects on the binding to other macromolecules. To analyze this, when several protein chains are available in the 3D structure, a “+” icon will be activated on the top left corner of the panel. This icon allows displaying all chains present in the 3D-structure file. Chains that do not correspond to the protein of interest are displayed in ribbon and colored in green to differentiate them. When these additional chains are displayed, their residues can be automatically displayed if they are in the vicinity of a residue of the protein of interest on which the user double clicked (or after its selection from the sequence alignment or variant panels). In this case, the interactions between the residue of interest of the main chain and close residues of the other chains are also displayed for analysis. A simple or double click on a residue of the other proteins will zoom the representation on it. Of note, this will not change the focus of the multiple alignment and variant panels, which are dedicated only to the main chain (i.e., the selected protein of interest).

Other options and use controls are described in Supplementary Data file.

#### The sequence alignment panel

The sequence alignment of the human protein to several orthologs is displayed in the sequence alignment panel situated at the bottom of the page (Fig. 5b). Importantly, when a 3D structure is displayed in the corresponding panel, the residues present in that structure are automatically numbered according to the human numbering, as defined by the reference UniProt sequence. Of note, all residues in our list of PDB files were renumbered to correspond to the UniProt-based numbering.

A single click on the residue number or a double click on a residue letter in the sequence alignment will center the 3D structure and the variant panels accordingly.

#### Other pages

Information regarding data collection and curation are provided in the FAQ page accessible through the header menu. A video showing the usage of the website is also available in the video page. Should users need more assistance, a contact form is available.

### Conclusion and perspectives

Structural information is key to the interpretation of protein variants. Swiss-PO has been designed to be used interactively in preparation or during MTBs where key aspects for decisions support are made available for the medical oncologist, pathologist, or biologist. As such, this web service is expected to gain rapid momentum in our community and to help the complex therapeutic choices required to guide precision oncology.

The database of Swiss-PO is expected to grow in the future releases to cover the FoundationOne panel of 324 genes^28^. We will also add an additional source of protein structures, notably high-quality structural models generated by Swiss-Model^29^,^30^. We will also complement the variant panels with other sources of information, including the SVIP-O^13^ and ClinVar^5^ databases.

## Methods

Data retrieved from reliable sources such as UniProt/Swiss-Prot^11^, the PDB^4^, and the CKB CORE database^24^ were used and manually curated to create Swiss-PO databases. The current Swiss-PO database was developed using scripts written in Python3 and is managed using the open source relational database management system MySQL version 5.7.30. The sequence alignments were done with MUSCLE^26^. The visualization software UCSF Chimera (version 1.13.1)^31^ was used for the visual curation of the structures and sequence alignments. The website was written in HTML5, PHP (version 7.2.24), and JavaScript. All data regarding curated PDB files including a detailed description of their content, sequence alignments, and previously determined effects of known mutations are stored in an SQL database managed using the open source relational database management system MySQL version 5.7.30. Multiple sequence alignments are displayed using the MSAViewer JavaScript visualization tool^32^, version 1.0.3. Three-dimensional structures are shown using the NGL Viewer web application for molecular visualization^33^, version 2.0.0.

### Reporting summary

Further information on research design is available in the Nature Research Reporting Summary linked to this article.

## Supplementary information

Supplementary data file -Swiss-PO: a new tool to analyze the impact of mutations on protein three-dimensional structures for precision oncology

REPORTING SUMMARY

## Data Availability

The databases were build using publicly available data retrieved from the UniProt/Swiss-Prot^11^, the PDB^4^, and the CKB CORE database^24^, with the exception of proteins DDR2, EGFR, and PIK3CA whose data were generously provided by The Jackson Laboratory from the CKB BOOST database.
