# Supplementary material for: Swiss-PO: a new tool to analyze the impact of mutations on protein three-dimensional structures for precision oncology
Source: NPJ Precis Oncol. 2021 Mar 18;5:19. doi: 10.1038/s41698-021-00156-5 (PMC7973488; doi:10.1038/s41698-021-00156-5)
Supplement: Supplementary file 1 — Supplementary data file -Swiss-PO: a new tool to analyze the impact of mutations on protein three-dimensional structures for precision oncology [file 41698_2021_156_MOESM1_ESM.pdf]

Swiss-PO: a new tool to analyze the impact of mutations on protein three-dimensional structures for precision oncology.

Fanny S Krebs[1,‡], Vincent Zoete[1,2,‡,*], Maxence Trottet[1,2], Timothée Pouchon[2], Christophe Bovigny[2], Olivier Michielin[1,2,3,*].

1 Computer-aided molecular engineering,Department of Oncology, Ludwig Institute for Cancer Research, University of Lausanne, Lausanne, Switzerland

2 Molecular Modelling Group, Swiss Institute of Bioinformatics (SIB), Lausanne, Switzerland

3 Department of Oncology, Ludwig Institute for Cancer Research, University Hospital of Lausanne, Lausanne, Switzerland

‡: these authors contributed equally to this work.

*: correspondence to vincent.zoete@unil.ch and olivier.michielin@chuv.ch

Supplementary Information

Databases Preparation

Identifying sequence differences between the protein present in a PDB file and the reference protein

To reinforce the structure quality, we used the Basic Local Alignment Search Tool (BLAST)[1], provided by UniProt[2] and implemented in the Bio.Blast package from Biopython v.1.73[3], to align the full human amino acid sequence of the protein retrieved from the UniProt database with the one corresponding to each chain of the same protein in the structures, which gave us the sequence identity between both of them.

Structure file of protein complexes

For each structure, a file was created by merging all chain components' coordinates (e.g., relevant ligands, antibodies, etc.) related to the chain of interest. This file corresponds to the initial structure displayed on the website visualization section. When the structure involves other subunits, like another protein or an antibody, a second file was created. The latter gathered all the other subunits coordinates and their ligands, and constitutes the quaternary version of the corresponding structure.

Scoring structure files

Based on the visualization of the structures and on the above-mentioned information, a score between 1 and 10 was given to each of them. This score is used to rank the proteins in the structure table based on their relevance for the interpretation of mutation. A score of 10 was given to the structure that will be automatically uploaded when a protein is selected, and which was selected as the most representative one for a given protein. When possible, this structure corresponds to the wild-type protein in complex with a well-known FDA approved drug. When no structure fulfilled these conditions, we selected the one with the most representative domain of the protein or one with an FDA approved drug. Criteria for attributing a score to each 3D structure are summarized in Supplementary Table 5.

Selection of the orthologous protein sequences

UniProt/UniProtKB provides two types of amino acid sequences: (i) the reviewed ones from the SwissProt database that are manually curated based on the literature and curator-evaluated computational analysis, and (ii) the unreviewed ones from the TrEMBL database, which are computationally analyzed and are waiting for full manual annotation. For each gene, we applied manual control of the sequences potentially included in the Swiss-PO database. For all genes, we retrieved the reviewed human sequence and those of the nine orthologues, including reviewed and unreviewed ones, when available. Then, for a given gene, and for each of the nine organisms, all retrieved orthologous sequences were aligned with the reviewed corresponding human sequence. This step allowed selecting, for each organism, the sequence that shows the best coverage and alignment with the human amino acid sequence. The alignment was done with the MUltiple Sequence Comparison by Log-Expectation tool (MUSCLE)[4] package present in Biopython v.1.73[3]. When several sequences were available for an organism, without the possibility to select a particular one on a rational basis, we kept them all, and added a note to inform the user. We chose to keep an unreviewed sequence rather than a reviewed one when the alignment or the coverage of a relevant protein domain was more comprehensive. Sequences were also rejected if they create numerous gaps in the alignment, making the visualization and analysis of the latter particularly difficult.

Website Description

Other options for the selection panel

The selection panel contains two icons linking to the UniProt[2] and Nextprot[5] pages of the selected protein.

A "Quick search" box allows selecting directly the protein of interest and centering the variant panel on the mutated residue, or the closest ones if no mutation has been reported previously for that residue in the CKB or Swiss-Prot databases. This "Quick search" box takes a mutation as an input, following the Human Genome Variation Society recommendations for the description of protein sequence variants[6] (https://varnomen.hgvs.org).

Other options of the variant panel

A “magnifying glass” icon in the header of the “Variant” column allows focusing the variant table directly on a residue or a mutation of interest thanks to a drop-down menu listing all known mutations of the protein, or a search box taking a residue number as an input.

3D panel user controls

Users can control the positioning of the system using the following commands:

- Mouse left-button + drag: rotation
- Mouse right-button + drag: translation
- Mouse wheel up and down: zoom in and out
- Hitting "i" key: rolling the system. Hitting "i" again stops the rolling.
- Hitting "k" key: rocking the system. Hitting "k" again stops the rocking.

Hovering the mouse pointer on an atom will display the atom name, as well as the residue or ligand name and its number above the 3D viewer.

A reset button on the bottom right corner of the panel allows cancelling all positioning, zooming and interaction displaying.

Full screen display can be obtained by clicking on the corresponding icon on the top left corner of the panel.

Clicking on the information icon, in the top left corner opens a pop-up box containing a summary of the information available for the displayed 3D structure: PDB code, experimental method used to determine the structure, quality metrics, content in terms of the principal chain (corresponding to the protein of interest) as well as other chains and ligands, the total fraction of the protein covered by this 3D structure with a graphical representation of the corresponding sequence regions, missing residues, mutations and insertions. Additional remarks, regarding the nature of the protein domain for instance, are also provided. As for the protein-selection panel, ligands present in the chosen 3D structure are listed according to their usual name if they are approved or experimental drugs, or following the 3-character coding of the PDB. In both cases, a link is provided to the PDBeChem ligand dictionary to get more information on this

small molecule. Similarly, other chains present in the 3D structures are identified by their 1-character code in the structure file (which is also displayed in the 3D panel upon hovering the mouse pointer on its atoms). The nature of the chain is also given, either in plain text (e.g. antibody) or as a UniProt code. The latter is clickable and linked to the corresponding page of the UniProt web site.

Supplementary Table 1: List of the 50 genes and the corresponding proteins, included in the Ion AmpliSeq™ Custom Cancer Hotspot Panel

Genes	Uniprot code	Names
ABL1	P00519	Tyrosine-protein kinase ABL1
AKT1	P31749	RAC-alpha serine/threonine-protein kinase
ALK	Q9UM73	ALK tyrosine kinase receptor
APC	P25054	Adenomatous polyposis coli protein
ATM	Q13315	Serine-protein kinase ATM
BRAF	P15056	Serine/threonine-protein kinase B-raf
CDH1	P12830	Cadherin-1
CSF1R	P07333	Macrophage colony-stimulating factor 1 receptor
CTNNB1	P35222	Catenin beta-1
DDR2	Q16832	Discoidin domain-containing receptor 2
EGFR	P00533	Epidermal growth factor receptor
ERBB2	P04626	Receptor tyrosine-protein kinase erbB-2
ERBB4	Q15303	Receptor tyrosine-protein kinase erbB-4
EZH2	Q15910	Histone-lysine N-methyltransferase EZH2
FBXW7	Q969H0	F-box/WD repeat-containing protein 7
FGFR1	P11362	Fibroblast growth factor receptor 1
FGFR2	P21802	Fibroblast growth factor receptor 2
FGFR3	P22607	Fibroblast growth factor receptor 3
FLT3	P36888	Receptor-type tyrosine-protein kinase FLT3
GNA11	P29992	Guanine nucleotide-binding protein subunit alpha-11
GNAQ	P50148	Guanine nucleotide-binding protein G(q) subunit alpha
HNF1A	P20823	Hepatocyte nuclear factor 1-alpha
HRAS	P01112	GTPase HRas
IDH1	O75874	Isocitrate dehydrogenase [NADP] cytoplasmic
IDH2	P48735	Isocitrate dehydrogenase [NADP] mitochondrial
JAK2	O60674	Tyrosine-protein kinase JAK2
JAK3	P52333	Tyrosine-protein kinase JAK3
KDR	P35968	Vascular endothelial growth factor receptor 2
KIT	P10721	Mast/stem cell growth factor receptor Kit
KRAS	P01116	GTPase KRas
MAP2K1	Q02750	Dual specificity mitogen-activated protein kinase kinase 1
MET	P08581	Hepatocyte growth factor receptor
MLH1	P40692	DNA mismatch repair protein Mlh1
MPL	P40238	Thrombopoietin receptor
NOTCH1	P46531	Neurogenic locus notch homolog protein 1
NPM1	P06748	Nucleophosmin
NRAS	P01111	GTPase NRas
PDGFRA	P16234	Platelet-derived growth factor receptor alpha
PIK3CA	P42336	Phosphatidylinositol 4,5-bisphosphate 3-kinase catalytic subunit alpha isoform

PTEN	P60484	Phosphatidylinositol 3,4,5-trisphosphate 3-phosphatase and dual-specificity protein phosphatase PTEN
PTPN11	Q06124	Tyrosine-protein phosphatase non-receptor type 11
RB1	P06400	Retinoblastoma-associated protein
RET	P07949	Proto-oncogene tyrosine-protein kinase receptor Ret
SMAD4	Q13485	Mothers against decapentaplegic homolog 4
SMARCB1	Q12824	SWI/SNF-related matrix-associated actin-dependent regulator of chromatin subfamily B member 1
SMO	Q99835	Smoothened homolog
SRC	P12931	Proto-oncogene tyrosine-protein kinase Src
STK11	Q15831	Serine/threonine-protein kinase STK11
TP53	P04637	Cellular tumor antigen p53
VHL	P40337	von Hippel-Lindau disease tumor suppressor

Supplementary Table 2: Number of PDB and coordinate files, including chains, ligands, peptides, treated for the 3D database (3D-DB).

Genes	PDB retrieved	Chains	Ligands	Peptides	Selected for the 3D-DB
ABL1	65	116	190	41	61
AKT1	32	35	85	12	31
ALK	61	66	142	1	57
APC	26	48	82	11	20
ATM	5	7	0	0	5
BRAF	80	130	229	0	55
CDH1	16	30	114	15	12
CSF1R	20	26	89	4	19
CTNNB1	24	55	117	5	19
DDR2	3	21	3	8	2
EGFR	197	281	870	69	193
ERBB2	33	62	119	44	28
ERBB4	10	12	28	2	9
EZH2	17	50	84	7	16
FBXW7	5	11	41	6	5
FGFR1	62	103	279	0	52
FGFR2	43	107	266	4	39
FGFR3	5	8	12	0	5
FLT3	8	15	46	0	8
GNA11	1	4	3	1	1
GNAQ	1	1	0	1	1
HNF1A	2	4	4	4	2
HRAS	175	326	1205	18	173
IDH1	44	86	309	6	43
IDH2	8	14	74	6	6
JAK2	93	96	265	2	90
JAK3	35	36	119	0	35
KDR	50	61	197	12	50
KIT	23	34	90	8	17
KRAS	160	228	1149	44	111
MAP2K1	46	71	240	0	45
MET	84	116	197	21	80
MLH1	3	7	30	0	2
MPL	0	0	0	0	0
NOTCH1	24	81	154	16	23
NPM1	4	17	16	0	4
NRAS	5	6	21	1	4
PDGFRA	7	7	29	0	6
PIK3CA	51	108	134	0	50

PTEN	6	8	10	0	6
PTPN11	62	68	178	19	60
RB1	15	32	33	6	12
RET	27	79	397	8	25
SMAD4	11	26	62	14	10
SMARCB1	8	34	10	2	8
SMO	10	18	66	2	5
SRC	64	77	167	43	59
STK11	3	9	23	0	2
TP53	186	453	765	154	151
VHL	55	195	242	11	55
Total	1975	3485	8985	628	1772

Supplementary Table 3: PDB code and names of FDA approved drugs present in the 3D database.

PDB 3-letters codes	Usual names	PDB 3-letters codes	Usual names
0WN	Afatinib	XIN	Nintedanib
0WM	Afatinib	YY3	Osimertinib
3LU	Cefsulodin	LQQ	Palbociclib
4MK	Ceritinib	6ZZ	Ribociclib
5B4	Altiratinib	RAP	Sirolimus
69Q	Enasidenib	AY7	Asciminib
B2B	Tautomycetin	STU	Staurosporine
HKI	Neratinib	88Z	Foretinib
P01	Purvalanol	H8H	Saracatinib
P30	Quizartinib	5P8	Lorlatinib
VJK	Decernotinib	QB4	Lorlatinib
P06	Dabrafenib	53P	Lorlatinib
AXI	Axitinib	8E8	Ibrutinib
DB8	Bosutinib	G4K	Borussertib
VGH	Crizotinib	03P	TAK-285
1N1	Dasatinib	09L	Olaparib
YMX	Entrectinib	0RP	Torcetrapib
AQ4	Erlotinib	1C9	Dacomitinib
2RC	Fostamatinib	2TA	Fedratinib
IRE	Gefitinib	38O	Dovitinib
1E8	Imbruvica	3JD	Niraparib
STI	Imatinib	5HV	Darapladib
LEV	Lenvatinib	6T2	Crenolanib
NIL	Nilotinib	799	taselisib
RXT	Ruxolitinib	7AS	Odanacatib
BAX	Sorafenib	8RC	Naquotinib
B49	Sunitinib	949	Polmacoxib
K88	SU-6656	AV3	Cediranib
ZD6	Vandetanib	BA0	Zanubrutinib
032	Vemurafenib	C6F	gilteritinib
0LI	Ponatinib	IBI	Volasertib
MI1	Tofacitinib	LUR	Lumiracoxib
6ZV	Abemaciclib	MZJ	Evobrutinib
EMH	Alectinib	TIV	Tivantinib
6GY	Brigatinib	6FS	Rigosertib
EUI	Cobimetinib	P31	Pexidartinib
FMM	Lapatinib	5FS	Erdafitinib

Supplementary Table 4: PDB code and names of investigational drugs present in the 3D database.

PDB 3-letters codes	Usual names	PDB 3-letters codes	Usual names
ML9	PF-04691502	3E8	Tepotinib
919	Rebastinib	353	BMS-777607
55J	RAF-265	D7D	Abrocitinib
LCJ	GDC-0623	0XZ	Capivasertib
FE5	Sapanisertib	706	Motesanib
8JC	Rociletinib	AEE	AEE-788
YDJ	AZD-7762	CHU	RO-5126766 free base
07J	Infigratinib	V0L	Savolitinib
L5G	AMG-208	AV9	Tivozanib
1KS	Taladegib	L1X	Merestinib
LWJ	DEBIO-1347	63K	SAR-125844
AZ5	AZD-1480	IZG	TAK-733
2HB	Filgotinib	0RF	Ipatasertib
1LT	Alpelisib	VRA	Refametinib
BZI	Benzimidazole	3EW	Selumetinib
5SF	Erdafitinib	406	Bafetinib
SX8	SGX-523	4BM	PD-0325901
6S1	Miransertib	3K3	Lifirafenib
VIS	Vismodegib	TZ0	Futibatinib
3OR	RO-4987655	KRW	PF-04217903
66T	AZD-4547	3E8	Tepotinib
3E8	Tepotinib		

Supplementary Table 5: Criteria to attribute a score to each protein structure related to a given gene. Note that exception can occur depending on the structures available per protein. Redundancy also concerns similar chains from a unique PDB file (ex: chains B or C). In the latter case, the highest score chain was selected by alphabetical order, when all other criteria were equal.

Score	Ranking criteria
10	• Most representative structure available for the gene (e.g., high resolution, wild-type, presence of an FDA-approved ligand, important domain of the protein, etc.)<br>Only one structure is scored 10 per gene, and will appear by default when opening the “PDB file” panel
9	• Wild-type structures in presence of an FDA approved ligand
8	• Structures containing an FDA approved drug in the presence of a mutation
7	• Wild-type and apo structures
6	• Wild-type structures in presence of non-FDA approved ligands
5	• Mutated structures in presence of non-FDA approved ligands
4	• Mutated apo structures
3	• Structures redundant with those scored 8-10
2	• Structures redundant with those scored 5-7
1	• Structures redundant with ones scored 4

Supplementary Table 6: Organisms used for the sequences alignments analysis of each gene, taken from the NCBI reference sequence organisms.

Species names	Taxon names
Human	*Homo sapiens*
Chimpanzee	*Pan troglodytes*
Macaque	*Macaca mulatta*
Mouse	*Mus musculus*
Rat	*Rattus norvegicus*
Dog	*Canis lupus familiaris*
Bovine	*Bos taurus*
Chicken	*Gallus gallus*
Zebrafish	*Danio rerio*
Frog	*Xenopus tropicalis*

Supplementary Table 7: Number of amino acid sequences retrieved from UniProt and checked manually, per gene and in total.

Genes	Number of sequences checked	Number of sequences selected
All genes	768	478
ABL1	18	9
AKT1	15	9
ALK	10	7
APC	14	9
ATM	13	10
BRAF	20	10
CDH1	16	10
CSF1R	15	10
CTNNB1	12	10
DDR2	19	10
EGFR	21	9
ERBB2	10	8
ERBB4	23	10
EZH2	15	10
FBXW7	10	9
FGFR1	19	10
FGFR2	22	10
FGFR3	29	10
FLT3	12	8
GNA11	14	9
GNAQ	11	10
HNF1A	16	10
HRAS	12	8
IDH1	16	10
IDH2	10	9
JAK2	17	10
JAK3	15	9
KDR	13	10
KIT	13	8
KRAS	14	10
MAP2K1	17	10
MET	18	10
MLH1	12	10
MPL	16	7
NOTCH1	20	10
NPM1	20	10
NRAS	17	10
PDGFRA	16	10
PIK3CA	11	11

PTEN	12	9
PTPN11	17	10
RB1	18	10
RET	13	10
SMAD4	15	9
SMARCB1	15	10
SMO	13	10
SRC	18	10
STK11	11	9
TP53	13	9
VHL	12	10
Total	768	478

References

[1] Altschul SF, Gish W, Miller W, Myers EW, Lipman DJ. Basic local alignment search tool, J Mol Biol, 215, 403–10, (1990).

[2] Bateman A, Martin MJ, O'Donovan C, Magrane M, Alpi E, et al., UniProt: The universal protein knowledgebase. Nucleic Acids Res, 45(D1), D158-D169, (2017)..

[3] Cock PJA, Antao T, Chang JT, Chapman BA, Cox CJ, Dalke A, et al., Biopython: Freely available Python tools for computational molecular biology and bioinformatics. Bioinformatics, 25, 1422–3, (2009).

[4] Thompson JD, Plewniak F, Poch O. A comprehensive comparison of multiple sequence alignment programs. Nucleic Acids Res, 27, 2682–90, (1999).

[5] Gaudet P, Michel PA, Zahn-Zabal M, Britan A, Cusin I, et al., The neXtProt knowledgebase on human proteins: 2017 update, Nucleic Acids Res, 45, D177–82, (2017).

[6] den Dunnen JT, Dalgleish R, Maglott DR, Hart RK, Greenblatt MS, et al., HGVS Recommendations for the Description of Sequence Variants: 2016 Update, Hum Mutat, 37, 564–9, (2016).
